# Newborn Screening for X-Linked Adrenoleukodystrophy in Georgia: Experiences from a Pilot Study Screening of 51,081 Newborns

**DOI:** 10.3390/ijns6040081

**Published:** 2020-10-23

**Authors:** Patricia L. Hall, Hong Li, Arthur F. Hagar, S. Caleb Jerris, Angela Wittenauer, William Wilcox

**Affiliations:** 1 Department of Human Genetics Atlanta, Emory University, Atlanta, GA 30322, USA; hong.li@emory.edu (H.L.); alwitte@emory.edu (A.W.); william.wilcox@emory.edu (W.W.); 2 Georgia Department of Public Health, Atlanta, GA 30033, USA; arthur.hagar@dph.ga.gov; 3 EGL Genetics, Tucker, GA 30084, USA; sj253697@pcom.edu

**Keywords:** newborn screening, X-linked adrenoleukodystrophy, peroxisomal biogenesis disorders, very long chain fatty acids

## Abstract

We screened 51,081 newborns for X-linked adrenoleukodystrophy (ALD) using a two-tiered strategy quantifying very long chain lysophosphatadylcholines (LPC). Our testing strategy used flow injection tandem mass spectrometry for the first-tier analysis of LPCs, and second-tier quantification of C26:0 LPC using liquid chromatography tandem mass spectrometry. There were 364 specimens considered abnormal using our first-tier algorithm that relied on the four LPC measurements and post-analytical tools. Second-tier test results were reported as normal or abnormal based on a cutoff for the single analyte, C26:0 LPC. Eleven cases were reported as abnormal based on second-tier test results. One male with ALD was identified, and two females with peroxisomal biogenesis disorders were also identified. A single female case remains unresolved, due to a loss to follow up after a negative molecular test result for *ABCD1* gene sequencing. The positive predictive value for confirmed, clinically relevant disorders during this pilot study was 27.3%. Challenges identified during the study period were based around coverage for confirmatory testing, particularly if family members needed molecular testing, which is an ongoing issue with newborn screening in Georgia. We also encountered issues with the follow up for a patient who remained asymptomatic. Due to the different timelines involved with clinical findings in ALD, follow-up coordination may be more difficult, particularly if the child identified by newborn screening (NBS) is the only member of the family affected, or able to be tested.

## 1. Introduction

X-linked adrenoleukodystrophy (ALD, MIM #300100) is a progressive leukodystrophy caused by deficiency of the adrenoleukodystrophy protein, resulting in accumulation of very long chain fatty acids (VLCFA). The accumulation of these VLCFA damage the white matter and adrenal cortex [1]. ALD is inherited in an X-linked recessive manner, and is caused by pathogenic variants in *ABCD1*. This accumulation of VLCFA can result in varying clinical presentations in affected males, broadly defined as a childhood cerebral form, adrenomyeloneuropathy and isolated primary adrenal insufficiency (PAI). Affected males may progress between phenotypes, particularly if their initial presentation is PAI. Although the inheritance is described as X-linked recessive, many females who are heterozygous for *ABCD1* pathogenic variants will develop symptoms later in life. The onset of symptoms in affected males is variable. The childhood cerebral form most commonly presents between the ages of four and eight and may be initially mistaken as having attention deficit disorder/hyperactivity. The childhood cerebral form of ALD is rapidly progressive and can result in total disability within two years of initial symptoms. Treatment for ALD varies with age and disease progression. PAI is treated with appropriate corticosteroid replacement therapy. Males at risk for the childhood cerebral form of ALD are monitored to determine their need for hematopoietic stem cell transplantation (HSCT), which can reduce disease progression in affected boys [2,3].

Early corticosteroid replacement treatment in affected males can prevent adrenal crisis and death. For boys who are at risk of progressing to the childhood cerebral form of ALD, routine monitoring can provide an appropriate window for HSCT, which must be performed before advanced neurological involvement is evident. In many cases, when symptoms are recognized clinically, and a diagnosis is made, the disease has already progressed beyond the point where HSCT will be effective. This need for early identification and intervention has made ALD a target for newborn screening (NBS) programs. In 2015, ALD was recommended for inclusion on the Recommended Uniform Screening Panel (RUSP) in the United States [4,5]. The National Institutes of Health (NIH) have developed a strategy for funding states who wish to conduct pilot studies for disorders which have been recently added to the RUSP, and for those which are in advanced stages of consideration. The information obtained from these pilot studies allows states to make informed decisions about implementation timelines and strategies. 

In the United States, New York has been screening all infants for ALD since 2013 [5,6]. Several other states have also started screening after the inclusion of ALD on the RUSP [7]. Screening for ALD is accomplished using tandem mass spectrometry (MS/MS), with several different second-tier tests being utilized. The first-tier test quantifying long chain lysophosphatadylcholines (LPC) in dried blood spots is highly sensitive but lacks specificity. To reduce false positives, second-tier tests utilizing liquid chromatography have been developed [6,8]. First-tier quantification of LPCs is amenable to multiplexing with existing MS/MS NBS assays, including enzyme assays for lysosomal disorders [8] or amino acid and acylcarnitine analysis [9]. 

The addition of ALD to NBS programs is the first widespread adoption of a relatively common X-linked disorder, which brings additional challenges to follow up. While males with the childhood cerebral form of ALD are the primary target of screening, some heterozygous females will also be identified. When screening for autosomal recessive disorders, the likelihood of another affected family member is low, except in the cases of older, unscreened siblings. In a study of Fabry disease pedigrees, another X-linked disorder with varying clinical presentations, the identification of an affected proband resulted in an average of five other affected family members being identified [10]. Similar numbers can likely be expected for ALD, although the new mutation rate has varied in reports [11,12]. Diagnosing and caring for relatives is beyond the scope of most NBS systems, and needs to occur in specialty clinics. NBS programs should anticipate this scenario and have plans for appropriate referrals.

## 2. Materials and Methods 

We screened 51,081 specimens from newborns born in Georgia between 07 December 2017 and 12 June 2017. The study was reviewed by the Institutional Review Boards from Emory University and the Georgia Department of Public Health, and deemed exempt, as it was not considered research. After all NBS dried blood spot specimens received by the Georgia Department of Public Health Laboratory were punched for the standard NBS panel, they were punched for ALD testing and transported daily to EGL Genetics, where the assay was performed, a strategy previously used for a lysosomal storage disorder pilot study [13]. A two-tiered screening strategy was utilized, with post-analytical tools (Collaborative Laboratory Integrate Reports; CLIR) being used to analyze the first-tier flow injection MS/MS results. This post-analytical tool was set up to use the concentrations of C20, C22, C24 and C26 LPC, as well as the ratios between them. These tools utilize age-adjusted reference ranges, as well as confirmed cases to generate a score for the overall profile representing the likelihood that a screening result is abnormal. A result was considered abnormal by the first-tier test if it gave an informative score (>1st percentile of confirmed cases). Any results that were abnormal by the first-tier assay were referred for second-tier analysis by a liquid chromatography-MS/MS assay designed to reduce false positive results [8]. The second-tier test for ALD could be performed on the same sample, which reduced turnaround times for the results. The second-tier test results were reported out based on a cutoff, with a normal/abnormal result based on the measured value of C26:0 LPC (>0.30 nmol/mL was abnormal). This cutoff was established based on the analysis of normal specimens and dried blood spots from patients with confirmed peroxisomal disorders. For follow-up testing, all infants were referred for plasma VLCFA analysis. Based on the screening results and clinical presentations, molecular testing was pursued, including analysis of *ABCD1* or other genes involved in peroxisomal biogenesis disorders. During the pilot study period, testing was covered by the child’s insurance, as is standard for NBS follow up in Georgia.

## 3. Results

Using the first-tier screening and post-analytical tools, 364 screens out of 51,081 were referred for second-tier testing. Had a simple cutoff for C26:0 LPC been used at this stage (>0.30 nmol/mL), 2712 samples would have been referred for second-tier testing, which is a significant extra burden for instrument time and analysis. The CLIR post-analytical tool significantly reduced the referral rate to the second-tier test. After the second-tier testing was completed, 11 results were reported as abnormal. Seven of these were confirmed to be normal based on follow-up testing (false positives); one female outcome is pending, based on discrepant confirmatory test results. One male infant was molecularly confirmed to have ALD. Two female infants were identified to have other clinically significant peroxisomal biogenesis disorders. We did not identify any female infants who had abnormal VLCFA on confirmatory testing. Screening results for the 11 reported cases are shown in Table 1. Molecular testing was not pursued on all infants whose confirmatory plasma testing was reported to be normal. Studies have shown that some heterozygous females with normal plasma VLCFA can have elevated C26:0 LPC [14]. The positive predictive value (PPV) for ALD NBS during this pilot study was 9.1%. The PPV for all clinically relevant peroxisomal disorders (one ALD and two peroxisomal biogenesis disorders) was 27.3%. The incidence of ALD in Georgia was 1:51,081 births, which is at the lower end of the reported incidence of 1:20,000 to 1:50,000 [1]. The incidence of peroxisomal biogenesis disorders in Georgia, based on this study, was 1:25,540, which is higher than previous estimates [15,16]. There were no known false negative results during the study period; however, these potential cases may have not yet presented clinically. It is expected that some individuals who are heterozygous for *ABCD1* pathogenic variants may be missed due to being biochemically normal. 

Clinical findings and summaries for the four cases with abnormal confirmatory testing are shown in Table 2. The single confirmed case of ALD was an African American male, born to a 19-year-old mother, who was the first child in his family. He was clinically asymptomatic and his confirmatory VLCFA were abnormal and suggestive of ALD. Sequence analysis of *ABCD1* identified a hemizygous, pathogenic variant. Molecular testing had not yet been performed on at-risk family members. He has not attended follow-up appointments with endocrinology, nor obtained his scheduled MRI at one year of age despite extensive efforts from the clinic. There were two cases of peroxisomal biogenesis disorders identified during the study period. A Caucasian female had an abnormal NBS collected at 10 h of age, and plasma VLCFA were collected at the same time based on clinical findings. She had pulmonary hypertension, polymicrogyria, periventricular cystic structures, bilateral cystic kidneys, stippled humeral epiphyses and dysmorphic facies. Care was withdrawn at one day of life, and the results of confirmatory testing and NBS were reported after her death. Molecular testing ordered prior to her death identified two pathogenic variants in *PEX5*, consistent with the clinical diagnosis of Zellweger syndrome. The second case of a peroxisomal biogenesis disorder was also a Caucasian female, but with a much milder clinical presentation. At five weeks of age, the child had vomiting, failure to thrive and liver dysfunction. Confirmatory VLCFA were abnormal, and molecular testing identified two pathogenic variants in *PEX1*. A single unresolved case was an African American female, who had an abnormal NBS and abnormal plasma VLCFA on confirmatory testing. Sequencing and copy number analysis for *ABCD1* were both negative. At the time of initial evaluation, the plan was to follow up with repeat VLCFA to determine if the abnormalities persist. This testing has not been able to be obtained via coordination with the child’s primary care provider. She was asymptomatic at her last evaluation. In Minnesota’s initial cohort identified by NBS, they have also had an infant with suspected ALD, based on VLCFA and pedigree analysis, who had no causative molecular variant identified [7]. In such cases, a mild peroxisomal disorder cannot be ruled out completely.

## 4. Discussion

NBS for ALD in Georgia was able to identify individuals affected with ALD and other peroxisomal biogenesis disorders, without a significant burden of false positive (FP) results. Our overall positive predictive value (PPV) was similar to that reported in Minnesota, when considering all screens that required additional patient contact. Their initial screens reported out as “borderline”, and were resolved with a repeat NBS collection (PPV of approximately 25% in both states) [7]. After initially identifying a cluster of FP results in a short time period, we started to repeat all samples that were abnormal for confirmation (from the original NBS card; an additional sample was not collected). This FP rate did not persist throughout the entire study, and no obvious explanation was found. NBS can provide more accurate estimates for incidence of rare diseases, although early reports can vary significantly due to short periods of screening. In Georgia, our incidence for ALD was lower than what had been previously estimated, while the incidence for peroxisomal biogenesis disorders was higher. Early screening results from Minnesota showed the opposite, with a significantly higher incidence for ALD and no cases of peroxisomal biogenesis disorders identified [7]. Both findings likely represent an artifact of small sample sizes; however, continued screening will determine if this reflects true differences in population.

Clinically, the infant with ALD was identified presymptomatically and was appropriately guided for follow up. At this time, the family has not followed up with care in our area, and no familial testing has been completed. ALD is the first NBS disorder where the primary condition added to the screening panel has a delayed onset and does not require immediate (<1 month of age) intervention. The first symptoms of adrenal insufficiency may not become obvious for more than a year; however, earlier presentations have been reported [17]. Coordination of care and conveying to families the importance of the diagnosis at such a busy time in their lives may prove challenging. Education plays a key role in prompting family compliance with appropriate follow up and improved outcomes. However, problems with transportation, time off for appointments in working families, mistrust of the medical system and denial, particularly when there is a lack of affected relatives and an apparently healthy child, are common issues faced in the care of patients with a variety of genetic disorders. Education and engagement of primary care physicians in the process of long-term follow up may promote better compliance with specialist follow up. Insurance coverage for familial testing, particularly for uninsured parents, remains a barrier to appropriate evaluation of at-risk family members. Coverage for the newborn’s VLCFA testing was not a significant hurdle during the study period. However, timely approvals for molecular testing was an issue, and testing for at-risk family members, particularly parents, was very challenging due to lack of health care coverage. Care coordination for patients identified through this pilot study program was managed through the existing network for peroxisomal patients in our Genetics Department.

ALD was nominated for inclusion on Georgia’s NBS panel in 2018, and the recommendation was approved by the Commissioner of Public Health. Funding was provided in the FY2020 budget. With the release of an FDA-cleared assay that includes very long chain LPCs alongside the analysis of amino acids and acylcarnitines (Perkin Elmer’s NeoBase2 kit), the barriers to implementing screening for ALD have lowered. After the implementation of a laboratory-developed test for the second-tier LC-MS/MS assay, screening was anticipated to begin in early 2020. Due to COVID-19-related delays, full population screening for ALD in Georgia began in May 2020, with a two-tiered testing strategy. During the first month of screening, another infant was diagnosed with a peroxisomal biogenesis disorder. One issue likely to persist with ALD screening in our state is the inability to reliably obtain appropriate testing for at-risk family members. Some states have utilized sequence analysis of *ABCD1* as a third-tier test to ensure timely access to sequencing results for follow up. This was not feasible in Georgia for the pilot study or full-time screening due to lacking infrastructure to perform such molecular testing in the newborn screening laboratory, and a lack of funding to perform it as a referral test.

## 5. Conclusions

A two-tiered strategy of NBS for ALD can effectively identify at-risk males presymptomatically, with performance characteristics that are similar to other conditions screened for using MS/MS assays. The second-tier LC-MS/MS assay was critical for reducing FP results to a manageable number, a key consideration when adding a disorder. A certain percentage of heterozygous females will also be identified, but the exact proportion of those who will be abnormal by the biochemical screening test is not precisely known. Our pilot study identified one affected male with ALD, and two females with peroxisomal biogenesis disorders, one severe and one more clinically mild. This incidence does not match what has been previously published about these disorders; however, it is a small sample size over a short screening period and will likely change over time. This information will likely become evident as screening expands to the population level. In addition to detecting ALD, screening programs and follow-up teams should be aware that they will likely identify peroxisomal biogenesis disorders and should have strategies in place to appropriately direct these children to care as well. In severe cases, the child may be symptomatic or deceased before NBS results are back. ALD is the most common X-linked disorder added to NBS and has significantly varied clinical presentations within families. Once a proband has been identified by NBS, states should consider strategies for testing at-risk family members and referring them for interventions where needed. This is particularly an issue for parents who may not have health care insurance coverage. The potential for routine identification of at-risk family members by NBS is an emerging situation and may require additional considerations for screening programs and follow-up teams. Performing sequence analysis of *ABCD1* in the NBS setting may reduce some health inequalities, but due to state regulations, public health funding would not be able to routinely pay for parental testing.

## Figures and Tables

**Table 1:** Screen-positive results identified by newborn screening (NBS) for adrenoleukodystrophy (ALD) in Georgia during the pilot study. PBD: peroxisomal biogenesis disorder.

Case ID	Birth Weight (g)	Gender	Age (hours)	NBS C24 (nmol/mL)	NBS C26 (nmol/mL)	2TT C26 (nmol/mL)	1TT CLIR Score (%ile)	Plasma VLCFA	Outcome
1	610	Female	655	0.48	0.62	0.44	42 (12)	Normal	Normal
2	3765	Male	37	0.63	0.81	0.74	100 (27)	Normal	Normal
3	2478	Female	25	0.47	0.67	0.37	159 (71)	Normal	Normal
4	3175	Female	514	1.74	1.75	1.34	234 (100)	Normal	Normal
5	2535	Male	25	0.54	1.11	0.88	234 (100)	Abnormal	ALD
6	3110	Female	24	0.45	0.58	0.44	141 (62)	Abnormal	Unresolved
7	3770	Male	25	0.61	0.53	0.46	159 (71)	Normal	Normal
8	2860	Female	26	0.70	0.78	0.60	234 (100)	Normal	Normal
9	2970	Male	2	0.57	1.08	0.83	227 (89)	Normal	Normal
10	2730	Female	36	0.32	0.91	1.00	114 (41)	Abnormal	PBD
11	2740	Female	10	0.38	1.22	1.13	114 (41)	Abnormal	PBD

**Table 2:** Summary of individuals with abnormal confirmatory test results identified during this study.

Case ID	Gender	Race	Plasma VLCFA	Gene	Variant 1	Variant 2	Diagnosis	Current Status
5	Male	African American	Abnormal (C26:0 = 4.24 nmol/mL) *	*ABCD1*	c.1203delG	--	ALD	Lost to Follow up
6	Female	African American	Abnormal (C26:0 = 1.27 nmol/mL) **	*ABCD1*	None	None	Unresolved	Lost to Follow up
10	Female	Caucasian	Abnormal (C26:0 = 6.26 nmol/mL) *	*PEX1*	c.2097dupT	C.2528 G>A (p.Gly843Asp)	PBD	Followed by multiple services
11	Female	Caucasian	Abnormal (C26:0 = 6.44 nmol/mL)	*PEX5*	c.583 C>T (p.Gln195Ter)	c.1279 C>T (p.Arg427 Ter)	PBD	Deceased

All variants were reported as pathogenic by the performing laboratory. * reference range for C26:0<1.30. ** reference range for C26:0 0.17–0.73. Case IDs are the same as used in Table 1.
